# Lumbar Plexus Disorders Presenting as Low Back Pain: A Narrative Review of a Practical Diagnostic Imaging Approach

**DOI:** 10.3390/diagnostics16162509

**Published:** 2026-08-09

**Authors:** Federico Bruno, Raffaele Natella, Giorgio Reverchon, Giulia Pacella, Michela Bruno, Mario Brunese, Giulio Lombardi, Corrado Caiazzo, Marcello Zappia

**Affiliations:** 1 Department of Biotechnological and Applied Clinical Sciences, University of L’Aquila, 67100 L’Aquila, Italy; 2 Department of Medicine and Health Science “V. Tiberio”, University of Molise, 84100 Campobasso, Italy; 3 Fondazione Trotula de Ruggiero, 84121 Salerno, Italy; 4 Biomedical Research Centre, Gruppo Forte, 84124 Salerno, Italy; 5 Department of Radiology, “St. Giuseppe Moscati” Hospital of National Relevance and High Specialty, 83100 Avellino, Italy; 6 Department of Precision Medicine, University of Campania “L.Vanvitelli”, 80138 Napoli, Italy

**Keywords:** diabetic amyotrophy, diagnostic approach, low back pain, lumbar plexopathy, lumbar plexus, magnetic resonance neurography, MRI, nerve imaging, psoas hematoma, retroperitoneal tumors

## Abstract

Low back pain (LBP) is one of the most common causes of medical consultation worldwide and is typically attributed to degenerative spinal disorders. However, a subset of patients presents with LBP related to extra-spinal conditions, including disorders of the lumbar plexus. These conditions are often underrecognized and may mimic lumbar radiculopathy, leading to delayed diagnosis or inappropriate management. The lumbar plexus, formed by the anterior rami of L1–L4 nerve roots, lies deep within the psoas muscle and can be affected by a wide range of pathological processes, including traumatic, compressive, inflammatory, infectious, and neoplastic conditions. A targeted literature search of the PubMed/MEDLINE database was performed to identify relevant English-language articles on lumbar plexus disorders, their imaging features, and diagnostic strategies published from inception to June 2026; representative imaging examples were selected from the co-authors’ clinical archives. The review summarizes the relevant anatomy, clinical presentation, and key imaging features of the most common plexus pathologies, including retroperitoneal tumors, psoas hematomas, inflammatory plexopathies, and iatrogenic injuries. Advances in imaging techniques, particularly magnetic resonance imaging (MRI) and magnetic resonance neurography (MRN), have significantly improved the ability to visualize the lumbar plexus and detect pathological changes involving peripheral nerves and surrounding structures. Additionally, a practical diagnostic imaging approach is proposed to assist radiologists and clinicians in recognizing lumbar plexus involvement in patients with atypical low back pain.

## 1. Introduction

Low back pain (LBP) is one of the most prevalent musculoskeletal conditions worldwide and represents a leading cause of disability and healthcare utilization. Recent estimates indicate that more than 600 million individuals were affected by LBP in 2020, with the burden expected to increase further due to population aging and growth [1].

In clinical practice, LBP is most attributed to degenerative spinal disorders, including intervertebral disc disease, facet arthropathy, spinal stenosis, and lumbar radiculopathy. Consequently, the diagnostic evaluation of LBP patients is usually directed to the lumbar spine [1] but not all low back pain is caused by disease of the spine. Some of the patients have symptoms that cannot be fully explained by degenerative or compressive spinal pathology. Extraspinal etiologies like retroperitoneal masses, inflammatory neuropathies, traumatic injuries, and metabolic disorders may be the underlying cause in these settings but are often missed in routine clinical evaluation [2,3].

Disorders of the lumbar or lumbosacral plexus are an important but underrecognized cause of low back and lower limb pain among these conditions. The lumbar plexus originates from the anterior rami of the L1–L4 nerve roots, with occasional contribution from T12 and is located deep within the psoas muscle in the retroperitoneal space and gives rise to the major peripheral nerves that supply the pelvis and lower extremity [2].

The lumbar plexus has complex neural connections and anatomic location which may cause clinical symptoms similar to lumbar radiculopathy such as low back pain, groin pain, anterior thigh pain, sensory changes, and motor deficits [2,3].

Various pathological processes may involve the lumbar plexus, including traumatic injuries, retroperitoneal tumors, inflammatory and diabetic radiculoplexus neuropathies, compressive lesions such as psoas hematomas, and iatrogenic complications. Many patients presenting with low back pain associated with groin or anterior thigh pain undergo imaging evaluation limited to lumbar spine MRI. While this approach is generally sufficient, it can miss disease in the retroperitoneal space or pelvic compartment. Disease processes that are not well appreciated on routine spinal imaging may involve the lumbar plexus and its proximal branches, particularly within the psoas compartment [3].

With the advent of imaging techniques, especially magnetic resonance imaging (MRI) and magnetic resonance neurography (MR neurography), it has become possible to directly visualize peripheral nerves and identify pathological changes involving the lumbar plexus and surrounding structures [3,4]. These techniques allow for the detection of nerve signal abnormalities, structural changes, and secondary muscle denervation changes, allowing for more accurate diagnoses in patients with atypical presentations [3,4,5].

The aim of this narrative review is to provide a practical overview of lumbar plexus disorders presenting as low back pain, with particular emphasis on imaging findings and diagnostic strategies. In addition, a structured imaging approach is proposed to assist radiologists and clinicians in recognizing lumbar plexus involvement and differentiating it from more common spinal causes of LBP.

Rather than reporting a systematic case series, this review provides a literature-based synthesis illustrated with original, previously unpublished imaging examples, selected to demonstrate the practical application of the proposed diagnostic approach in real clinical scenarios.

## 2. Materials and Methods

A targeted literature search was performed using the PubMed/MEDLINE database to identify relevant English-language articles up to June 2026. The search terms used included the combinations of lumbar plexus, lumbar plexopathy, lumbosacral plexopathy, low back pain, magnetic resonance imaging, MR neurography, retroperitoneal tumors, psoas hematoma, diabetic amyotrophy, traumatic plexopathy, extraforaminal disc herniation, infectious plexopathy, IgG4-related disease, and iatrogenic plexopathy.

Original articles, review articles, and selected case reports focusing on imaging findings, clinical presentation, and diagnostic strategies were considered. References of the retrieved articles were also screened to identify additional relevant publications.

The literature was narratively synthesized without formal quality assessment or meta-analysis, consistent with the objectives of a narrative review. Representative imaging examples were selected from the co-authors’ clinical archives to illustrate the principal imaging findings and support the proposed practical diagnostic approach.

## 3. Anatomy and Imaging of the Lumbar Plexus

### 3.1. Lumbar Plexus Anatomy

The lumbar plexus is created by the conjunction of the anterior rami of the L1–L4 spinal nerve roots with an occasional contribution from T12. It is situated in the posterior compartment of the psoas major muscle, in the retroperitoneal space, and gives rise to multiple peripheral nerves that supply motor and sensory innervation to the abdominal wall, pelvis, and lower extremity. The terminal subdivisions of the plexus include the iliohypogastric nerve, ilioinguinal nerve, genitofemoral nerve, lateral femoral cutaneous nerve, femoral nerve, and obturator nerve. These nerves arise from different parts of the psoas muscle and track through different routes anatomically as they cross toward the abdominal wall, sciatic indentation, and lower limb. Grasping this anatomy is crucial for radiologists when elucidating the findings of pelvic and retroperitoneal imaging, specifically in the setting of neuropathic pain syndromes and clinically suspected plexopathies [3].

### 3.2. Imaging Anatomy

**Figure 1:**
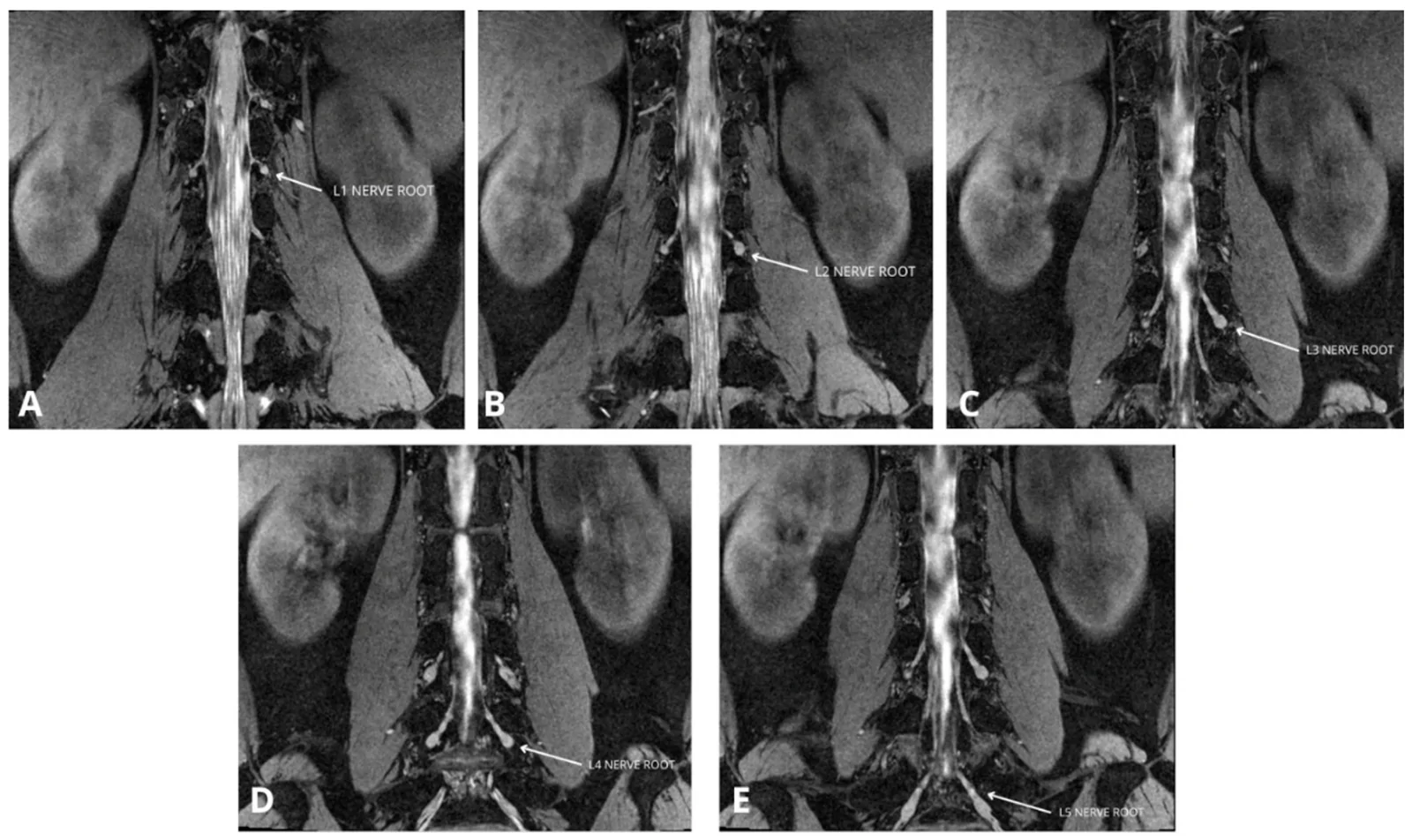
Coronal fat-suppressed T2-weighted MR neurography of the lumbar plexus in a healthy subject, demonstrating sequential identification of the individual nerve roots contributing to the plexus: L1 (**A**), L2 (**B**), L3 (**C**), L4 (**D**), and L5 (**E**). Original Image.

The lumbar plexus can be evaluated on magnetic resonance imaging (MRI), which is the primary modality for peripheral nerve imaging in the retroperitoneum. The plexuses are visualized as linear or fascicular structures with intermediate to mildly hyperintense signal on T1-weighted images and fluid-sensitive sequences, especially fat-suppressed T2-weighted or STIR images. MR neuroradiology sequence and parameters enhance nerve visualization by fat and vascular signal suppression [5]. Anatomically, the lumbar plexus is located in the posterior aspect of the psoas major muscle, where the nerve roots merge and create the principal branches of the plexus. The psoas muscle constitutes the radiological landmark that identifies the plexus. The posterior relation of the plexus is the quadratus lumborum muscle, which is part of the posterior abdominal wall musculature and serves as a separating muscle between the plexus and paraspinal muscles. The lateral and anterior relation of the plexus is inside the retroperitonium, where the plexus is related to the kidney, ureter, and the major vascular structure. The branches of the nerves reach the pelvic region inferiorly, where they cross the inguinal ligament and pelvis sidewall en route to the lower limb. This knowledge is important for recognizing the pathological conditions that may imply or compress the lumbar plexuses, such as retroperitoneal masses, inflammatory conditions, or trauma [6,7]. Figure 1 demonstrates an example of lumbar plexus imaging. 

## 4. Clinical Presentation: Low Back Pain Mimics Lumbar Plexus Disorders

Disorders of the lumbar plexus often present in a similar fashion to common degenerative spinal conditions and often result in initial diagnostic workup directed to the lumbar spine. Patients often present with low back pain that is associated with pain in the groin or anterior thigh, implying involvement of the femoral, genitofemoral or lateral femoral cutaneous nerve. Sensory symptoms, such as paresthesia and numbness, may involve the anterior thigh or pelvic region. These are often associated with motor deficits, especially in cases of femoral nerve involvement, such as weakness of knee extension. These manifestations are very similar to lumbar radiculopathy, and lumbar plexopathies are often under-recognized in the initial diagnostic work-up [8]. Pain that does not follow a single dermatome distribution or multiple peripheral nerve distributions should raise suspicion for pathology outside of the lumbar spine, including lumbar plexus disorders. If the lumbar spine MRI is negative or cannot explain the patient’s symptoms, other diagnoses involving the retroperitoneum or pelvis should be entertained. If clinical features such as disproportionate groin pain, anterior thigh pain, or neurologic deficits not confined to a single nerve-root distribution are present, the lumbar plexus should be evaluated further. In these situations, directed imaging of the retroperitoneal space may reveal occult lesions not seen on routine lumbar spine studies [3].

## 5. Imaging Techniques for Lumbar Plexus Evaluation

### 5.1. Conventional Radiography

Conventional radiography has an intrinsically limited role in the evaluation of lumbar plexus disorders, as it does not allow direct visualization of peripheral nerves or the retroperitoneal soft tissues. Its contribution is generally restricted to the assessment of osseous abnormalities, fractures, degenerative changes, or gross skeletal deformities that may provide indirect clues to the underlying pathology. Consequently, radiography is not considered a primary imaging modality for the assessment of lumbar plexopathy, and current diagnostic evaluation relies predominantly on cross-sectional imaging techniques, particularly CT and MRI [3,4].

### 5.2. MRI

**Figure 2:**
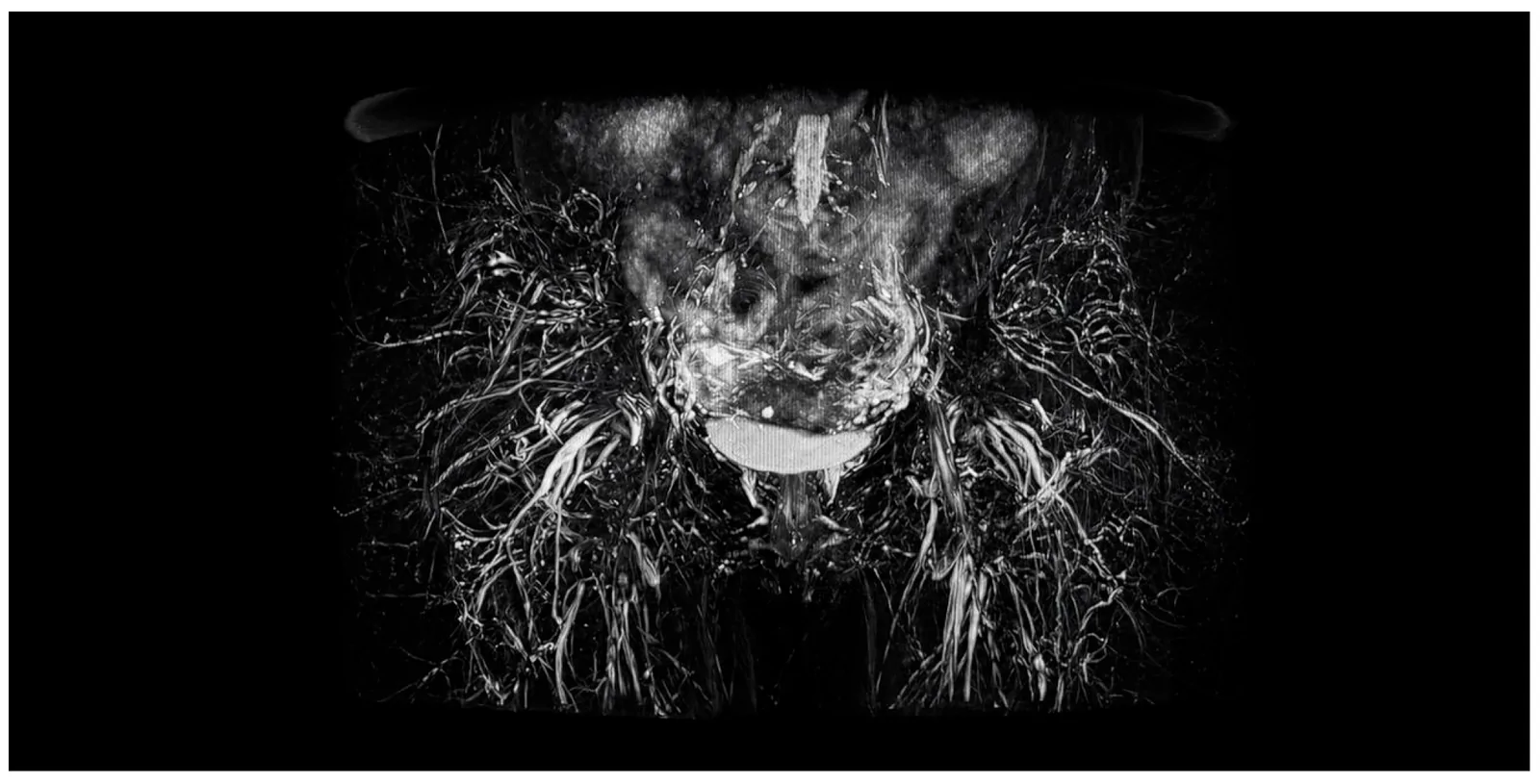
Three-dimensional maximum-intensity-projection (3D-MIP) MR neurography of the lumbosacral plexus in a healthy subject, demonstrating the fine fascicular detail and normal symmetric branching pattern of the nerve roots and their peripheral divisions bilaterally. Original Image.

MRI is the modality of choice to evaluate the lumbar plexus because of its superior soft-tissue contrast and excellent depiction of peripheral nerves and their adjacent retroperitoneal structures. T1-weighted sequences are helpful for anatomic detail and for evaluation of chronic denervation of muscle. Fat-suppressed T2-weighted or STIR sequences are very sensitive for nerve edema and for inflammatory change. Diffusion-weighted imaging (DWI) and MR neurography can provide additional benefits in peripheral nerve visualization [3]. MRI findings suggestive of a lumbar plexopathy include nerve enlargement, increased T2 signal intensity, and abnormal contrast enhancement after administration of gadolinium. Further clues to the diagnosis and to differentiate plexopathy from other causes of low back pain and lower limb symptoms may be obtained from additional signs, such as muscle denervation, edema in the acute phase, and chronic fatty infiltration on T1-weighted [9,10]. Figure 2 demonstrates an example of 3D MIP MR Neurography.

### 5.3. CT

While MRI remains the reference standard for direct evaluation of the lumbar plexus, computed tomography (CT) can play a complementary role in acute clinical settings thanks to its high spatial resolution and rapid acquisition that make CT the preferred first-line modality for detecting retroperitoneal hemorrhage, pelvic fractures, postoperative complications, and space-occupying lesions involving the psoas compartment [11].

CT accurately comprehends lesion size, extent, calcifications, and relationships with adjacent organs and vascular structures, facilitating lesion characterization and treatment planning. When imaging findings suggest possible lumbar plexus involvement, MRI or MR neurography should be considered for further evaluation of neural structures and associated denervation changes [12].

### 5.4. Ultrasound

Ultrasound plays a limited but complementary role in the assessment of lumbar plexus disorders. It may be helpful in the assessment of the femoral nerve, which can be evaluated with high-frequency linear transducers to assess caliber, fascicular architecture, and focal abnormalities. Ultrasound is also widely used to guide diagnostic and therapeutic procedures such as femoral nerve blocks and targeted perineural injections [13,14]. Compared to landmark-based techniques, real-time visualization improves the accuracy of needle placement and reduces the risk of vascular or neural injury. Ultrasound guidance has therefore become the standard of care for several diagnostic and therapeutic procedures involving the femoral nerve [14].

## 6. Lumbar Plexus Disorders Presenting as Low Back Pain

### 6.1. Traumatic Injuries

Traumatic injury of the lumbar plexus may result from high-energy pelvic trauma or iatrogenic injury following surgical procedures involving the pelvis, lumbar spine, hip, or retroperitoneum. Pelvic fractures, penetrating trauma, postoperative hematomas, and traction injuries represent the most common mechanisms of plexus damage. A psoas hematoma, particularly in anticoagulated patients, may produce secondary compressive neuropathy [15,16].

CT is usually the first-line imaging modality in the acute setting because of its rapid ability to detect pelvic fractures, retroperitoneal hemorrhage, and associated traumatic injuries. MRI allows for a better assessment of neural structures with evidence of nerve displacement, compression, increased T2 signal intensity, and secondary effects of muscle denervation such as acute edema and chronic fatty infiltration [8,17].

Recognition of these imaging findings is important because traumatic or compressive lesions affecting the lumbar plexus may clinically mimic lumbar radiculopathy and therefore require careful radiologic assessment of the retroperitoneal compartment and pelvic structures [3]. **Clinical examination:** Patients commonly present with weakness of hip flexion and knee extension, reduced or absent patellar reflex, and sensory loss over the anterior thigh and medial leg, reflecting femoral nerve involvement. Manual muscle testing and comparison with the contralateral limb help distinguish plexus injury from isolated musculoskeletal trauma [15,17].

### 6.2. Compressive Disorders

#### 6.2.1. Psoas Hematoma

A psoas or iliopsoas hematoma represents a compressive cause of lumbar plexopathy, as the lumbar plexus lies within the posterior portion of the psoas muscle in the retroperitoneal space. Expansion of a hematoma within the psoas compartment may therefore produce mechanical compression of the lumbar plexus or its branches, most commonly affecting the femoral nerve [18].

Iliopsoas hematomas most frequently occur in patients receiving anticoagulant therapy, particularly heparin or oral anticoagulants, although they may also develop following blunt trauma, vascular injury, spinal surgery, or other retroperitoneal interventions. Less commonly, spontaneous hematomas occur in patients with underlying coagulation disorders [19,20].

Patients typically present with acute or progressively worsening low back, groin, or anterior thigh pain associated with femoral neuropathy. Progressive nerve compression may result in quadriceps weakness, reduced patellar reflexes, and sensory disturbances involving the anterior thigh and medial leg [17].

Imaging plays a central role in diagnosis. CT is the preferred initial modality in emergency settings, demonstrating enlargement of the psoas muscle and hyperattenuating blood collections. MRI is particularly useful for evaluating lumbar plexus compression and associated denervation changes when neurological involvement is suspected [3].

**Figure 3:**
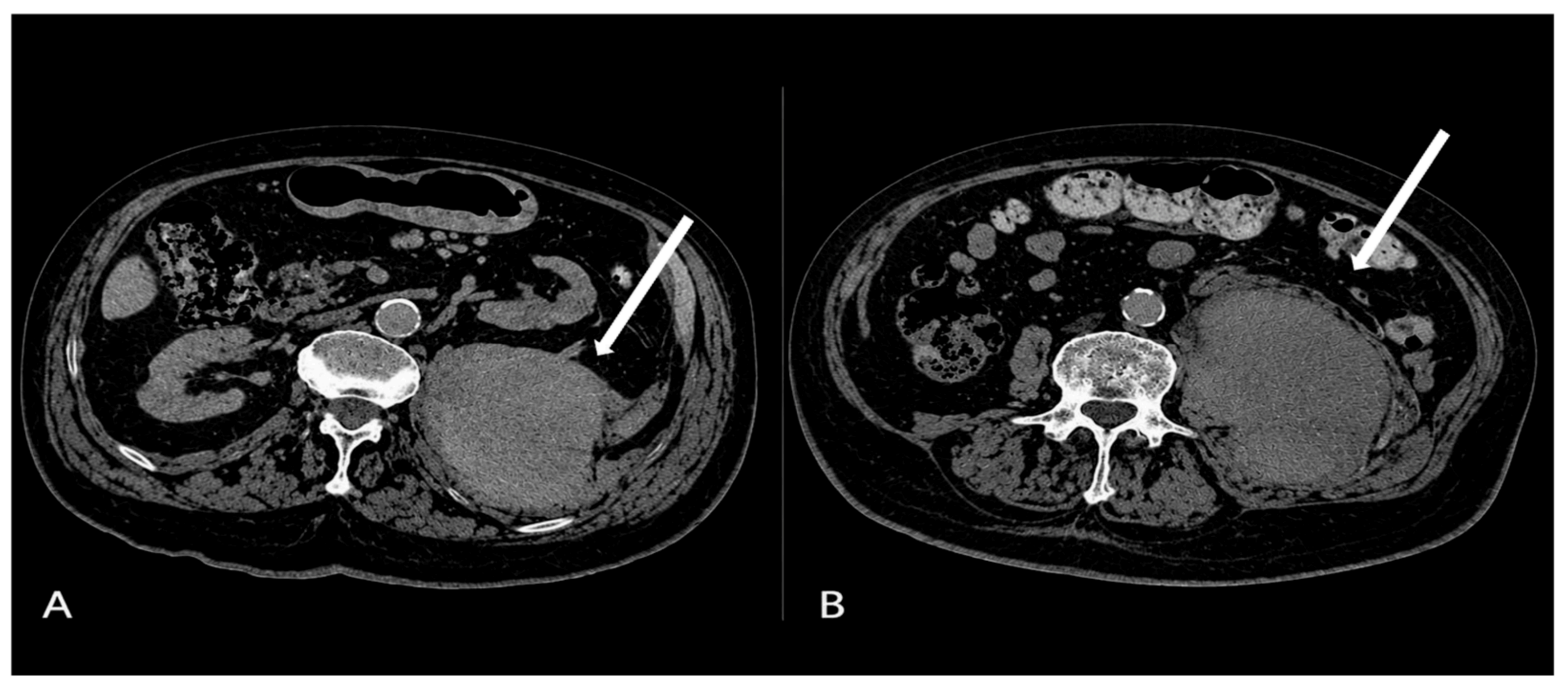
Case 1. A 76-year-old man receiving oral anticoagulant therapy presented with acute left-sided low-back and groin pain, progressive quadriceps weakness, and a reduced left patellar reflex. Axial contrast-enhanced CT images obtained at adjacent levels (**A**,**B**) demonstrate a large hematoma (white arrows) expanding the left psoas muscle, in close relationship with the expected course of the lumbar plexus, potentially accounting for the patient’s symptoms. Original Image.

An example of a compressive hematoma is shown in Figure 3.

**Clinical examination:** Pain is typically exacerbated by passive hip extension (psoas stretch test) and resisted hip flexion. Patients often adopt a flexed hip posture to reduce discomfort. Femoral neuropathy may manifest with quadriceps weakness and diminished patellar reflex [18,19,20].

#### 6.2.2. Retroperitoneal Tumors

**Figure 4:**
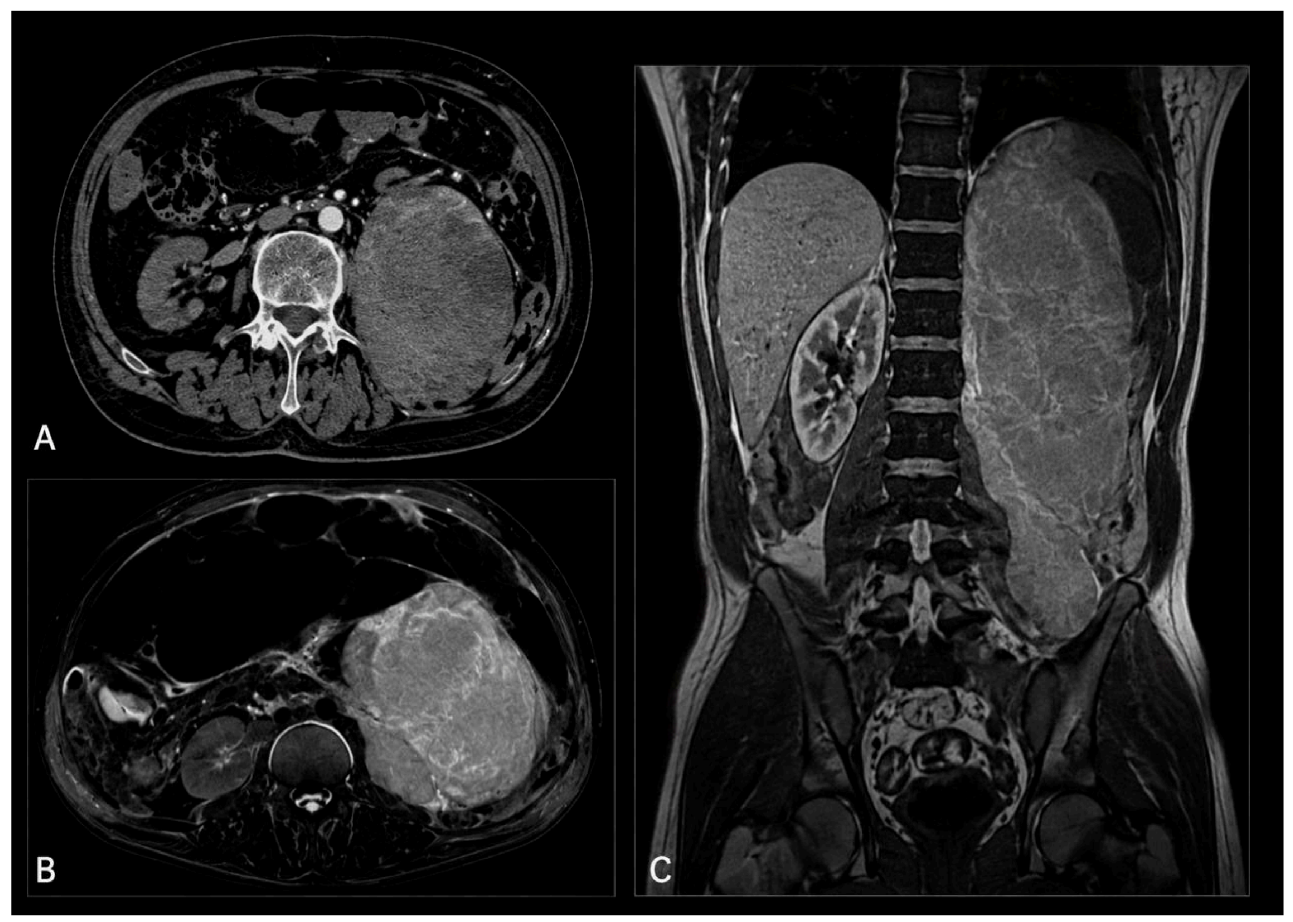
Case 2. A 56-year-old man presented with a several-month history of progressive left-sided low-back and flank pain radiating to the groin and anterior thigh. Axial contrast-enhanced CT (**A**) and axial T2-weighted MRI (**B**) demonstrate a large, well-circumscribed left retroperitoneal mass abutting the psoas muscle and the expected course of the lumbar plexus. Coronal T2-weighted MRI (**C**) depicts its full craniocaudal extent and relationship with the left kidney. Original Image.

Retroperitoneal tumors represent a cause of compressive lumbar plexopathy, as the lumbar plexus lies within the retroperitoneal space and in close relationship with the psoas muscle. Masses arising in this compartment may directly involve or compress the plexus or its branches, producing symptoms that may mimic lumbar radiculopathy [21]. Retroperitoneal neoplasms include lymphoma, primary retroperitoneal sarcomas, and metastatic disease. These lesions may compress, displace, or directly infiltrate the lumbar plexus, producing symptoms that frequently mimic lumbar radiculopathy [12]. Primary retroperitoneal sarcomas, lymphoma, and metastatic disease may compress, displace, or directly infiltrate the lumbar plexus. Metastatic involvement commonly arises from colorectal, gynecological, urological, or other pelvic malignancies [12]. CT provides excellent assessment of lesion size, local extension, and involvement of adjacent organs, whereas MRI offers superior soft-tissue characterization and evaluation of direct neural invasion. MR neurography may further demonstrate nerve enlargement, abnormal T2 signal intensity, contrast enhancement, and secondary denervation changes in affected muscles [3,19]. Recognition of these imaging patterns is important because retroperitoneal tumors involving the lumbar plexus may clinically present with low back pain, groin pain, or anterior thigh symptoms, often leading initially to evaluation focused on the lumbar spine rather than the retroperitoneal compartment [8]. An example of a retroperitoneal tumor is shown in Figure 4.

**Clinical examination:** Progressive symptoms, nocturnal pain, unexplained weight loss, and neurologic deficits in more than one peripheral nerve distribution should arouse suspicion of a retroperitoneal neoplasm. Weakness extending beyond a single myotome is particularly suggestive of plexus involvement [21].

#### 6.2.3. Extraforaminal Disc Herniation

Extraforaminal (or far-lateral) lumbar disc herniation represents an uncommon but clinically important cause of nerve compression that may mimic lumbar plexopathy. Unlike typical posterolateral disc herniations, extraforaminal disc protrusions occur lateral to the neural foramen, where the exiting nerve root travels close to the psoas muscle and the retroperitoneal compartment. Clinically, extraforaminal disc herniations can mimic lumbar plexopathy due to their anatomical proximity to the lumbar plexus and psoas compartment, especially when pain radiates into the groin or anterior thigh [22]. Radicular pain, sensory deficit, and muscle weakness related to a specific nerve root are frequently associated with extraforaminal disc herniation. However, the symptoms may be atypical, simulating lumbar plexus disorders due to the proximity of the nerve to the peripheral nerves forming the plexus structure [23]. MRI is the imaging modality of choice for detecting extraforaminal disc herniations, especially when dedicated sequences cover foraminal and extraforaminal regions. Common findings are focal disc protrusion lateral to the neural foramen with compression or displacement of the exiting nerve root and associated inflammatory changes. Careful assessment of these regions is essential because extraforaminal herniations may be overlooked on routine lumbar spine examinations [23,24].

**Clinical examination:** Unlike lumbar plexopathy, symptoms usually follow a single dermatomal and myotomal distribution. Straight leg raise or femoral nerve stretch tests may reproduce radicular pain depending on the affected level, helping differentiate radiculopathy from plexopathy [24].

### 6.3. Inflammatory Plexopathy

Inflammation or infection can trigger lumbar plexopathy without compression, often appearing like nerve root irritation in the lower back. These conditions may result from metabolic disorders such as diabetes mellitus, immune-mediated neuritis, or infectious processes involving the retroperitoneum or peripheral nerves [25].

Among these entities, DLRPN (diabetic amyotrophy) is the best-known inflammatory lumbosacral plexus plexopathy [26]. This disorder is thought to be caused by microvasculitis and immune-mediated ischemic injury of the peripheral nerves, leading to inflammation and axonal damage [27]. Patients with diabetic lumbosacral radiculoplexus neuropathy usually experience an acute or subacute onset of severe neuropathic pain in the lower back, hip, or anterior thigh, followed by progressive proximal weakness, muscle atrophy, and variable sensory deficits [28].

MRI typically shows nerve enlargement, increased T2 signal intensity, and contrast enhancement, which are consistent with inflammatory involvement of the lumbar plexus. Acute muscle denervation edema with chronic fatty atrophy of affected muscle groups is a secondary finding. Recognition of these imaging findings is important, as inflammatory plexopathies often mimic degenerative spinal disorders and need different therapeutic management strategies [3,4].

**Figure 5:**
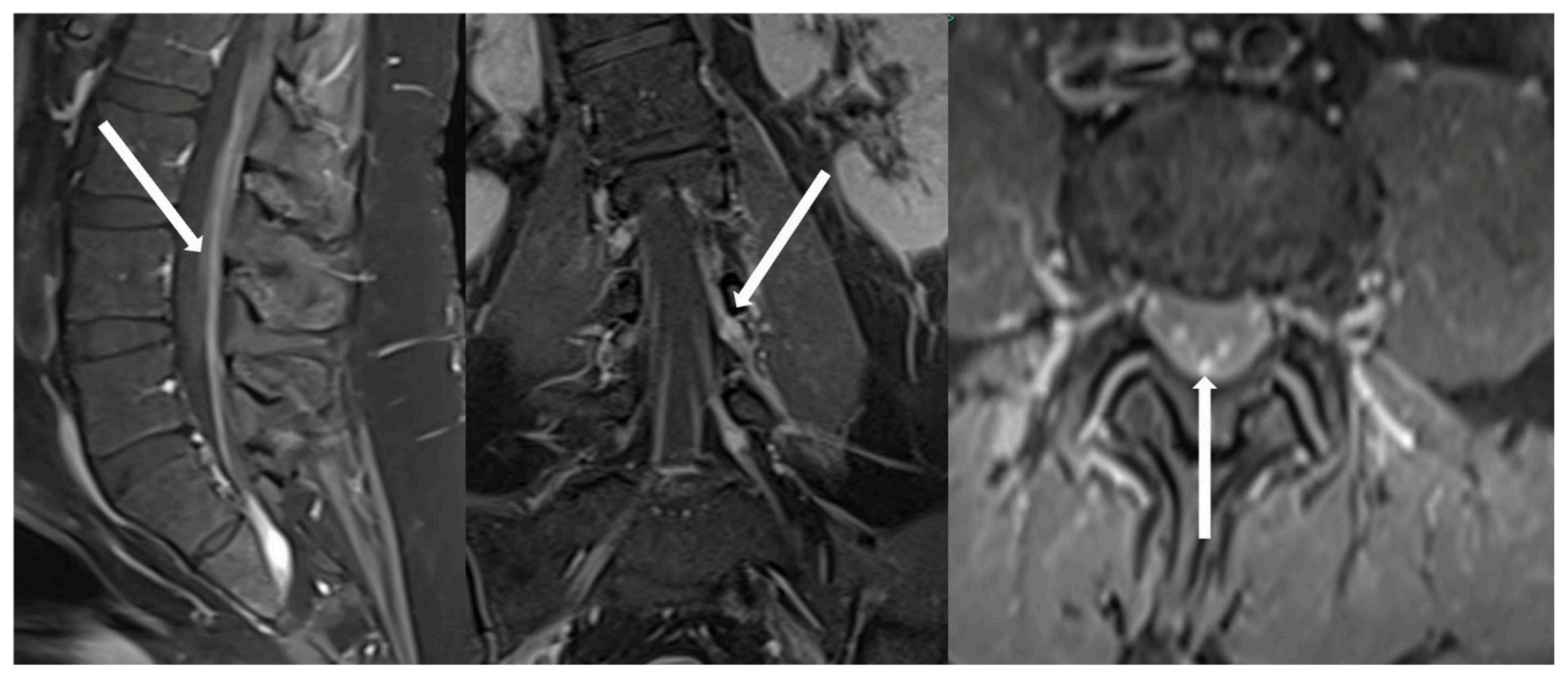
Case 3. A 52-year-old man presented with progressively worsening low-back pain and progressive symmetric lower-limb weakness and generalized areflexia. Contrast-enhanced fat-suppressed T1-weighted MRI in the sagittal, coronal, and axial planes demonstrates diffuse, smooth, and symmetric enhancement of the cauda equina nerve roots (white arrows), predominantly involving the ventral roots, contributing to the lumbosacral plexus. This pattern is suggestive of an inflammatory polyradiculoneuropathy, such as acute inflammatory demyelinating polyradiculoneuropathy, rather than an isolated lumbar plexopathy. Original Image.

**Figure 6:**
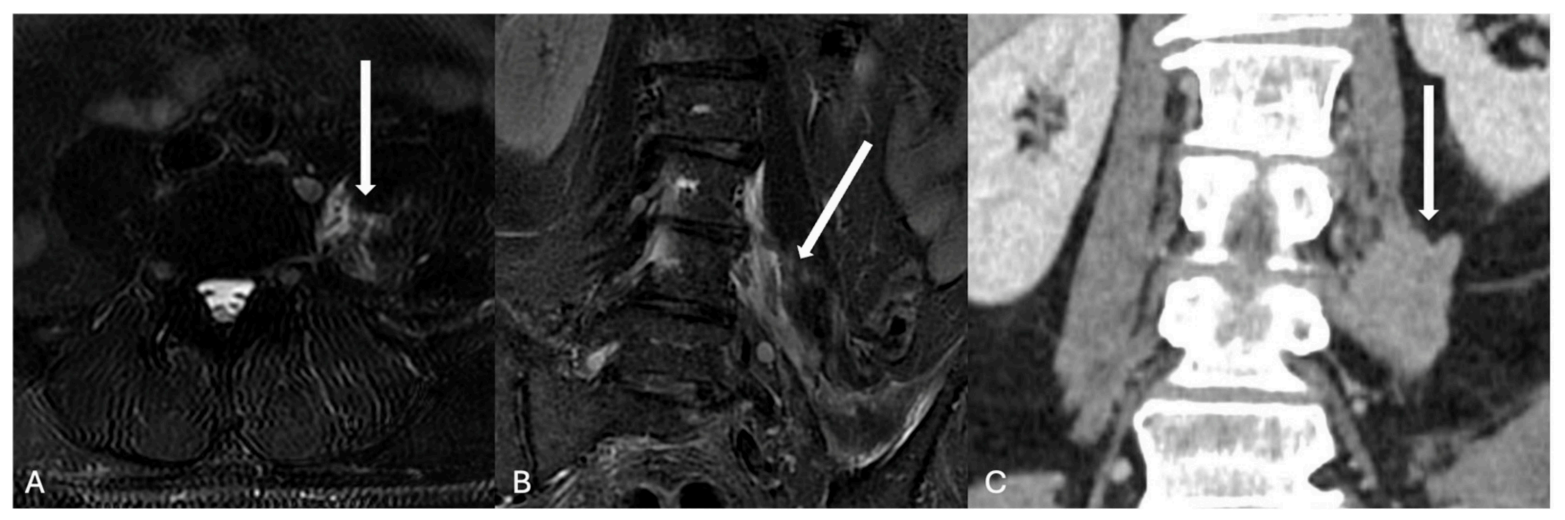
Case 4. A 66-year-old man presented with progressive left-sided low-back and groin pain radiating to the anterior thigh, associated with paresthesia and mild proximal lower-limb weakness. Axial fat-suppressed T2-weighted MRI (**A**) and contrast-enhanced fat-suppressed T1-weighted MRI (**B**) demonstrate infiltrative fibro-inflammatory tissue extending through the left paravertebral and iliopsoas compartments and encasing the lumbar plexus (white arrows). Coronal contrast-enhanced CT (**C**) confirms the retroperitoneal extension of the lesion. The final diagnosis was IgG4-related fibro-inflammatory disease. Original Image.

Representative MRI findings of an inflammatory polyradiculoneuropathy that may clinically mimic lumbar plexus disease are shown in Figure 5, demonstrating diffuse contrast enhancement of the cauda equina nerve roots. A chronic fibro-inflammatory variant, IgG4-related disease, may similarly involve the lumbar plexus through infiltrative encasement of the neural structures, closely mimicking a neoplastic process on imaging (Figure 6).

**Clinical examination:** Patients typically present with abrupt onset of severe unilateral thigh pain followed by progressive proximal weakness, muscle wasting, and weight loss. Weakness usually predominates over sensory loss and frequently involves multiple myotomes rather than a single nerve root [26,27,28].

### 6.4. Infectious Plexopathy

Infectious conditions can affect the lumbar or lumbosacral plexus, even if they occur less often than inflammation or pressure-related issues. Infections can involve the lumbar or lumbosacral plexus but are less common than inflammatory or compression problems. The most frequent cause of infectious plexopathy is contiguity of infection from adjacent retroperitoneal structures. Psoas abscesses can compress or directly invade the lumbar plexus and may present with low back pain, groin pain, femoral neuropathy, and progressive motor deficits. CT and MRI typically demonstrate enlargement of the psoas muscle, fluid collections, and surrounding inflammatory changes [29,30].

Varicella-zoster virus reactivation represents another recognized cause of infectious radiculoplexopathy. Although thoracic involvement is more common, lumbosacral disease may occur and typically presents with severe neuropathic pain followed by motor weakness. MRI may demonstrate nerve enlargement, increased T2 signal intensity, and contrast enhancement of the affected roots or plexus [4,31]. Recognition of these entities is important because infectious plexopathies may clinically mimic lumbar radiculopathy or other causes of low back pain, and appropriate imaging evaluation can facilitate early diagnosis and treatment [4].

**Clinical examination:** Fever, elevated inflammatory markers, local tenderness, and pain aggravated by hip movement should prompt suspicion of an infectious process. A thorough neurological examination is essential to document evolving motor deficits requiring urgent imaging [29,30,31].

### 6.5. Neoplastic Plexopathy

Neoplastic plexopathy most commonly results from metastatic disease, lymphoma, or direct extension of pelvic and retroperitoneal malignancies involving the lumbar plexus [32]. Tumor infiltration may occur through direct extension, perineural spread, or lymphatic dissemination within the retroperitoneum. Patients typically show up with progressive neuropathic pain followed by gradually worsening motor weakness and sensory deficits involving multiple peripheral nerve territories rather than a single nerve-root distribution [27].

**Figure 7:**
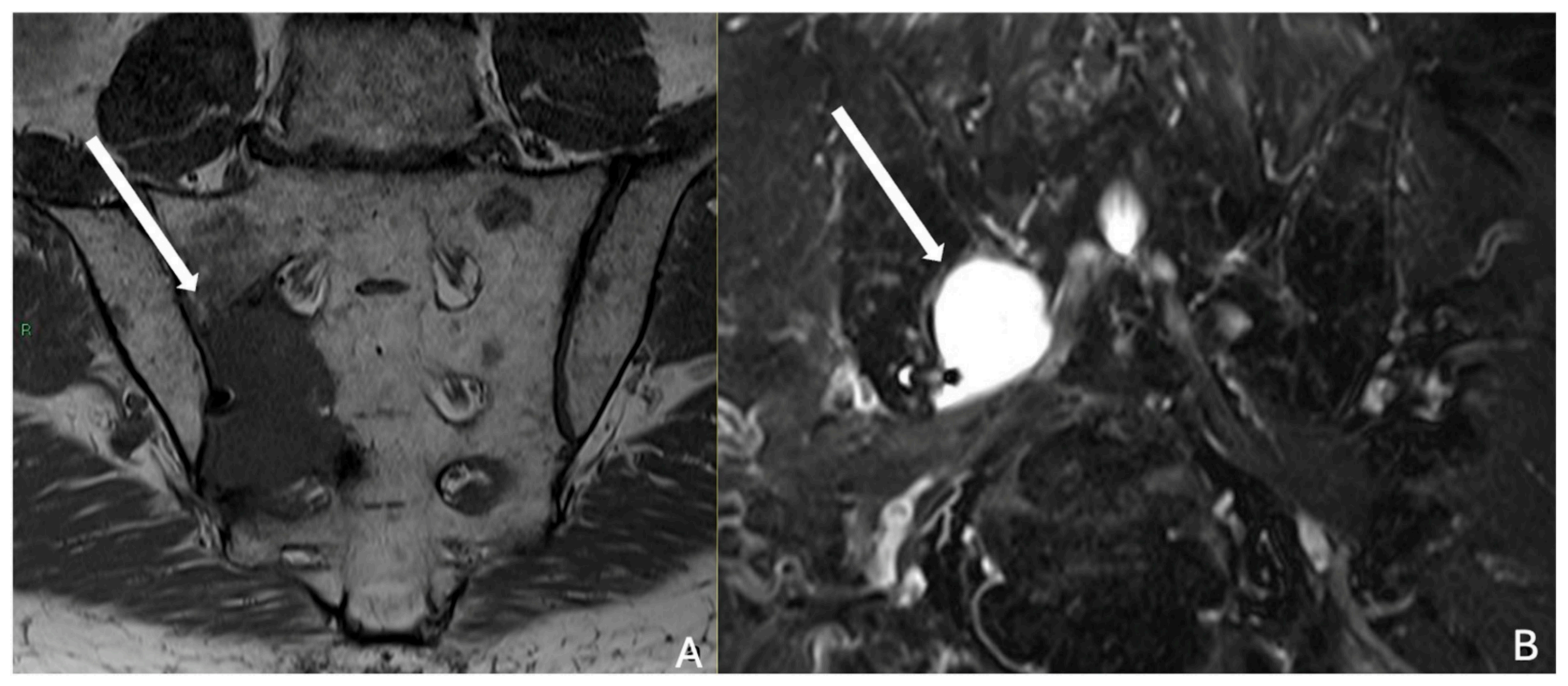
Case 5. A 68-year-old woman with known multiple myeloma presented with progressive right-sided lumbosacral pain, predominantly nocturnal, radiating to the right lower limb. Coronal T1-weighted pelvic MRI (**A**) demonstrates a focal marrow-replacing lesion within the right sacral ala (white arrow) . Axial fat-suppressed T2-weighted imaging (**B**) shows extraosseous soft-tissue extension involving the presacral region and adjacent sacral nerve roots of the lumbosacral plexus (white arrow). Original Image.

MRI and MR neurography are particularly useful for evaluating the lumbar plexus and detecting tumor involvement. Typical imaging findings include nerve thickening, abnormal contrast enhancement, and infiltration or encasement of the neural structures within the retroperitoneal space. Associated findings may include retroperitoneal masses, lymphadenopathy, or infiltration of the psoas muscle and adjacent pelvic structures [3,33]. MRI can also show secondary denervation changes in the muscle in the distribution of the affected nerves, which can further support the diagnosis of plexus involvement [5]. Recognition of these imaging findings is important because neoplastic plexopathy may be the initial presentation of an underlying malignancy or may represent disease progression in patients with known cancer [3,8]. Figure 7 shows a representative example of direct neoplastic infiltration, with a marrow-replacing lesion of multiple myeloma with extraosseous extension into the presacral region and involvement of the adjacent sacral nerve roots.

**Clinical examination:** Progressive pain, often worse at night and associated with unexplained weight loss or a history of malignancy, should raise concern for neoplastic plexopathy. Neurological deficits usually extend beyond a single root distribution and progressively worsen over time [32,33].

### 6.6. Iatrogenic Plexopathy

Iatrogenic injury represents an important cause of lumbar or lumbosacral plexopathy and may occur following a variety of surgical or interventional procedures involving the spine, pelvis, or retroperitoneal compartment. Because of the anatomical proximity of the lumbar plexus to the psoas muscle and major vascular structures, the plexus may be vulnerable to direct surgical trauma, postoperative hematoma, or traction injury during surgical manipulation [25]. Injuries of this type can also occur after lumbar spine surgery, particularly in surgeries that utilize a lateral transpsoas approach, where the surgical corridor must pass through the psoas muscle that contains the lumbar plexus. Furthermore, pelvic or retroperitoneal surgery, hip arthroplasty, and vascular procedures requiring the iliac vessels may also cause plexus injury due to direct nerve trauma, compression by postoperative collections, or ischemic injury [34,35].

Iatrogenic plexopathy can clinically manifest as postoperative low back pain, groin pain, or anterior thigh pain with associated motor weakness and sensory loss in the femoral or obturator nerve distribution. Symptoms may appear right after surgery or may develop gradually due to delayed complications such as hematoma formation or nerve compression by scar tissue [4]. Imaging is important in the evaluation of suspected postoperative plexopathy: MRI and MR neurography can show nerve thickening, signal changes, or denervation changes in the involved muscles, and CT may be helpful in identifying postoperative collections or hardware-related complications in the retro-peritoneal space [3,4]. Recognition of iatrogenic causes of lumbar plexopathy is important as early diagnosis may guide appropriate management and help distinguish postoperative complications from recurrent spinal pathology [5].

**Clinical examination:** Careful assessment of motor function, sensory deficits, and tendon reflexes immediately after pelvic, spinal, vascular, or hip surgery is essential. Early recognition of postoperative neurological deficits may facilitate prompt imaging and distinguish compressive lesions from inflammatory postoperative neuropathy [25].

## 7. Imaging Findings: Key Diagnostic Patterns and Structured Approach

Imaging findings can help refine the differential diagnosis of lumbar plexus disorders. MRI provides detailed anatomical assessment, while MR neurography further delineates nerve architecture and associated muscle changes. Typical imaging patterns include nerve thickening and increased T2 signal intensity, suggestive of inflammatory neuropathy; retroperitoneal masses involving the psoas compartment, suggestive of neoplastic plexopathy; psoas or retroperitoneal hematomas, indicative of compressive neuropathy; and muscle denervation edema, reflecting acute nerve injury or dysfunction [3,8]. Recognition of these patterns assists radiologists in differentiating inflammatory, compressive, traumatic, and neoplastic causes of lumbar plexopathy and guides further diagnostic evaluation [4,32] (Table 1).

In patients presenting with low back pain associated with groin or anterior thigh pain, diagnostic evaluation is typically focused on the lumbar spine, and lumbar spine MRI represents the most performed imaging test, given that degenerative spinal pathology and radiculopathy are the most frequent underlying causes [1]. When lumbar spine imaging is negative or inconclusive, evaluation should be extended to the psoas muscle and retroperitoneum, and advanced pelvic imaging, including MRI and, when indicated, dedicated MR neurography, should be considered if clinical suspicion persists. MR neurography enables direct visualization of the peripheral nerves and detection of nerve enlargement, abnormal signal intensity, or associated muscular denervation, findings suggestive of an infiltrative or inflammatory process. This stepwise approach can raise suspicion for retroperitoneal tumors, psoas hematomas, inflammatory plexopathies such as diabetic lumbosacral radiculoplexus neuropathy, or direct neoplastic or inflammatory nerve involvement, thereby guiding appropriate disease management [3,4,8].

**Figure 8:**
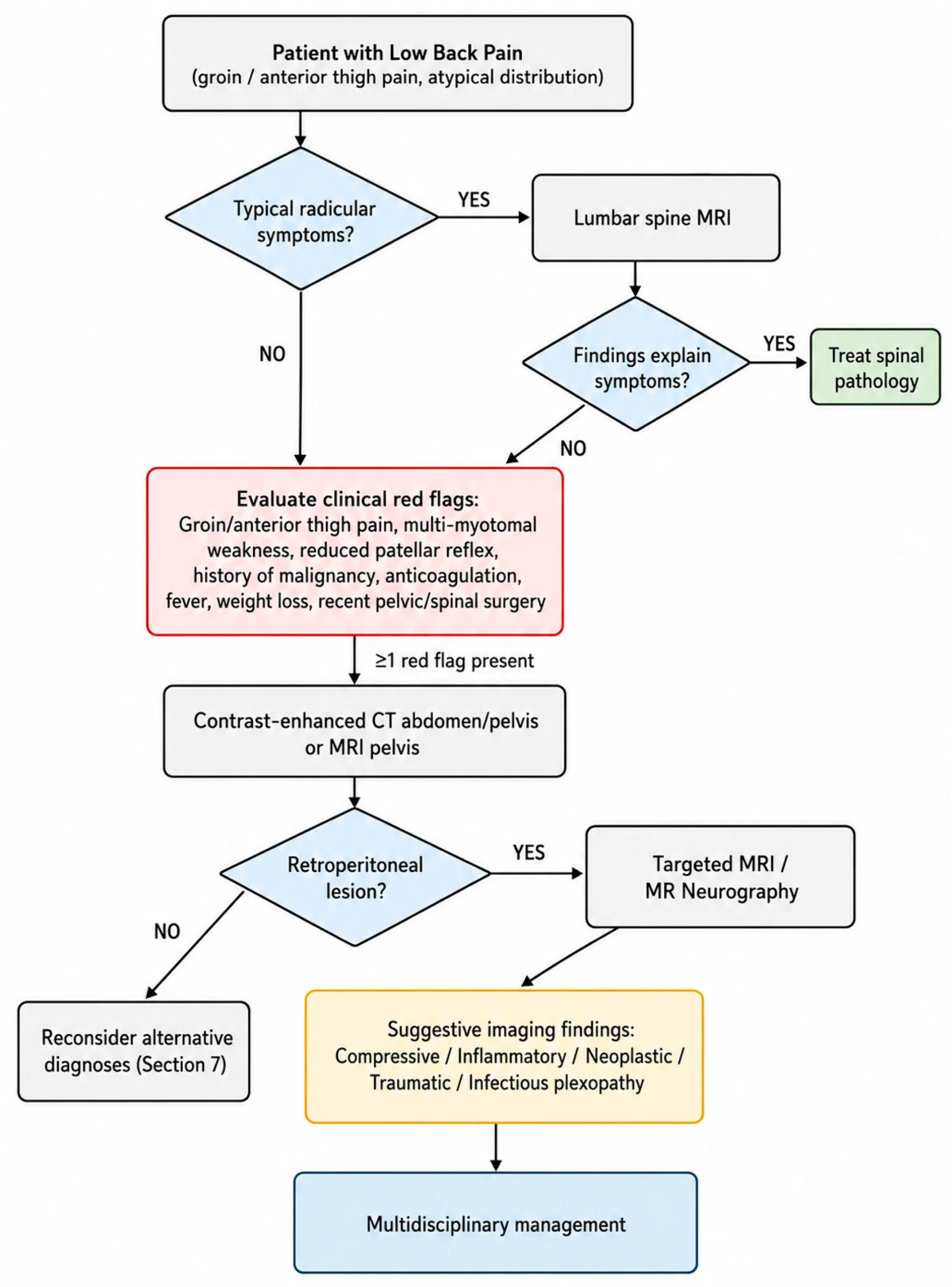
Proposed practical diagnostic imaging pathway for patients with low back pain and suspected lumbar plexus involvement. Clinical and imaging red flags (groin/anterior thigh pain, multi-myotomal weakness, reduced patellar reflex, history of malignancy, anticoagulation, fever, weight loss, recent pelvic/spinal surgery) prompt extension of imaging to the retroperitoneum and pelvis when lumbar spine MRI is negative or inconclusive.

As this is a narrative review, the proposed diagnostic pathway reflects a literature-based, non-validated approach rather than a clinically validated algorithm. Rather than introducing new imaging techniques, this approach optimizes their sequential use, integrating the clinical and imaging red flags summarized in Table 1 and the diagnostic pathway shown in Figure 8 to reduce diagnostic delay and avoid misclassification as degenerative spinal disease. Accurate differentiation from other conditions with overlapping presentations remains essential, as their management differs substantially: lumbar radiculopathy follows a dermatomal distribution corresponding to a single nerve root, with imaging evidence of nerve root compression on lumbar spine MRI [36]; peripheral neuropathy, such as diabetic polyneuropathy, typically presents with a distal symmetric pattern of sensory loss and weakness [37]; and hip joint pathology, including osteoarthritis, labral disease, or femoroacetabular impingement, produces pain that varies with joint movement and is evident on hip-focused imaging [38,39]. In contrast, lumbar plexopathy characteristically involves multiple nerve roots or their peripheral divisions, producing scattered motor and sensory findings that do not follow a single dermatomal pattern [3].

The proposed practical diagnostic imaging pathway, including the clinical and imaging red flags used to guide further evaluation, is summarized in Figure 8.

## 8. Conclusions

Lumbar plexopathy represents an underrecognized but clinically significant cause of low back, groin, and lower-limb symptoms, often overlooked because of its overlap with more common spinal disorders. Accurate diagnosis requires the combination of clinical evaluation and a multimodality imaging approach appropriate to the suspected underlying cause [2,5,6,18]; the correct identification of the plexopathy is crucial to avoid unnecessary spinal interventions, as evaluation of the retroperitoneum and pelvic structures can reveal inflammatory, neoplastic, or compressive plexopathies when lumbar spine imaging is not conclusive [36]. MRI is still the reference method for direct assessment of the lumbar plexus, whereas CT is particularly useful in emergency settings and for the detection of retroperitoneal hemorrhage, masses, or postoperative complications, and MR neurography is useful when conventional imaging is inconclusive or a detailed characterization of the nerves is needed [8,18,22,35]. This structured diagnostic approach, including careful evaluation of the retroperitoneum and targeted imaging of the lumbosacral plexus when indicated, may help radiologists and clinicians in recognizing lumbar plexus disorders, choosing the appropriate imaging strategies, and ultimately improving patient management [3,40]. As a narrative review, this work has several limitations that should be acknowledged. First, it does not follow the methodological framework of a systematic review, and the proposed diagnostic pathway reflects a descriptive, literature-based synthesis rather than a validated clinical decision tool; literature selection may therefore be subject to selection bias despite the structured search strategy adopted, and it does not include a systematic case series or clinical validation, although the illustrative figures derive from original, previously unpublished clinical cases contributed by the co-authors. Current evidence on lumbar plexopathy remains limited, consisting predominantly of case reports, small retrospective series, and expert opinion, reflecting the rarity and heterogeneous etiology of this condition, and imaging recommendations may vary according to institutional expertise, scanner availability, and access to advanced techniques such as MR neurography [6,22,35].

## Figures and Tables

**Table 1:** Clinical presentation, differential diagnostic clues, imaging strategy, and red flags for lumbar plexus disorders.

Condition			Key Clinical Presentation	Differential Clues	First-Line Imaging	Characteristic Findings	Complementary Imaging	Red Flag
Traumatic injury	Post-traumatic/postoperative LBP, groin or thigh pain, motor weakness	History of pelvic trauma or recent surgery	CT (acute)	Nerve displacement/compression within the psoas compartment; acute edema or chronic fatty atrophy of the innervated musculature		MRI/MR neurography		—
Psoas hematoma	Acute/progressive LBP, groin/thigh pain; quadriceps weakness; reduced patellar reflex	Anticoagulant therapy; recent trauma or surgery	CT (emergency)	Enlargement of the psoas muscle in the posterior retroperitoneal compartment, with hyperattenuating collection		MRI for compression/denervation		Anticoagulation
Retroperitoneal tumor	Progressive pain, multiterritory weakness, palpable mass, night pain, weight loss	History of malignancy; lymphoma, sarcoma, metastasis	CT/MRI	Mass abutting/infiltrating the psoas muscle within the retroperitoneal space; nerve enlargement and abnormal enhancement at the level of the lumbar plexus		MR neurography		Night pain
Extraforaminal disc herniation	Radicular pain, single dermatomal/myotomal distribution	Groin/thigh pain mimicking plexopathy	MRI (foraminal sequences)	Focal disc protrusion lateral to the neural foramen; nerve root compression at the extraforaminal/far-lateral zone		—		—
Inflammatory plexopathy (DLRPN)	Acute unilateral thigh pain, progressive proximal weakness, wasting, weight loss	Diabetes mellitus; immune-mediated microvasculitis	MRI/MR neurography	Nerve enlargement, T2 hyperintensity, and contrast enhancement of the lumbosacral plexus nerve roots		—		Weight loss
Infectious plexopathy	Fever, hip pain, local tenderness, progressive motor deficits	Psoas abscess; herpes zoster reactivation	CT/MRI	Enlargement of the psoas muscle with adjacent fluid collection; nerve enlargement/enhancement within the retroperitoneal compartment		—		Elevated CRP
Neoplastic plexopathy (infiltration)			Progressive pain, multiterritory deficits, night pain, weight loss	Known or suspected malignancy	MRI/MR neurography	Nerve thickening and abnormal enhancement with infiltration/encasement of the lumbar plexus within the retroperitoneal space, often involving the psoas muscle	CT for osseous involvement	History of cancer
Iatrogenic plexopathy			Postoperative LBP, groin/thigh pain, motor/sensory deficits	Recent pelvic, spinal, hip, or vascular surgery	MRI/MR neurography	Nerve thickening and signal abnormalities along the surgical corridor (e.g., transpsoas approach) or adjacent retroperitoneal/pelvic compartment, with denervation changes in the innervated musculature	CT for hardware/collections	—

## Data Availability

No research dataset was generated for this narrative review. No new data were created or analyzed in this study. Data sharing is not applicable to this article. Limited anonymized clinical information is reported solely to contextualize the illustrative imaging cases and is not publicly available due to patient privacy considerations.

## Abbreviations

The following abbreviations are used in this manuscript:

**Abbreviation**	**Full term**
CT	Computed Tomography
DLRPN	Diabetic Lumbosacral Radiculoplexus Neuropathy
LBP	Low Back Pain
MR	Magnetic Resonance
MRI	Magnetic Resonance Imaging
MRN	Magnetic Resonance Neurography
STIR	Short Tau Inversion Recovery
T1	T1-weighted (imaging sequence)
T2	T2-weighted (imaging sequence)
